# Effect of COPD severity and comorbidities on the result of the PHQ-9 tool for the diagnosis of depression: results from the COSYCONET cohort study

**DOI:** 10.1186/s12931-019-0997-y

**Published:** 2019-02-11

**Authors:** Sarah Marietta von Siemens, Rudolf A. Jörres, Jürgen Behr, Peter Alter, Johanna Lutter, Tanja Lucke, Sandra Söhler, Tobias Welte, Henrik Watz, Claus F. Vogelmeier, Franziska Trudzinski, Winfried Rief, Britta Herbig, Kathrin Kahnert, for the COSYCONET study group

**Affiliations:** 1 Institute and Outpatient Clinic for Occupational, Social and Environmental Medicine, Comprehensive Pneumology Center Munich (CPC-M), Ludwig-Maximilians-Universität München, Ziemssenstr. 1, 80336 Munich, Germany; 2 Department of Internal Medicine V, University of Munich (LMU), Ziemssenstr. 1, 80336 Munich, Germany; 3 Department of Medicine, Pulmonary and Critical Care Medicine, University Medical Center Giessen and Marburg, Philipps-University Marburg, Germany, Member of the German Center for Lung Research (DZL), Baldingerstrasse, 35043 Marburg, Germany; 4 Institute of Health Economics and Health Care Management, Helmholtz Zentrum München GmbH – German Research Center for Environmental Health, Comprehensive Pneumology Center Munich (CPC-M), Member of the German Center for Lung Research, Ingolstädter Landstr. 1, 85764 Munich, Germany; 5 ASCONET Study Coordination Office, University of Marburg, Baldingerstraße, 35043 Marburg, Germany; 6 Department of Pneumology, Hannover Medical School, Carl-Neuberg-Str. 1, 30625 Hannover, Germany; 7 Pulmonary Research Institute at LungenClinic Grosshansdorf, Airway Research Center North, Member of the German Center for Lung Research, Woehrendamm 80, 22927 Grosshansdorf, Germany; 8 Department of Internal Medicine V – Pulmonology, Allergology, Respiratory Intensive Care Medicine, Saarland University Hospital, Kirrberger Straße 1, 66424 Homburg, Germany; 9 Department of Clinical Psychology and Psychotherapy, Philipps-University Marburg, Germany, Gutenbergstraße 18, 35032 Marburg, Germany

**Keywords:** COPD, Depression, PHQ-9

## Abstract

The diagnosis of depression, a frequent comorbidity of chronic obstructive pulmonary disease (COPD), is often supported by questionnaires, such as the Patient Health Questionnaire 9 (PHQ-9). It is unknown to which extent its single questions are affected by the clinical characteristics of COPD patients.

We addressed this question in 2255 GOLD grade 1–4 patients from the COSYCONET (**CO**PD and **Sy**stemic **Co**nsequences - Comorbidities **Net**work) COPD cohort. The dependence on COPD severity was assessed using symptoms, exacerbation risk (GOLD A-D; modified Medical Research Council dyspnoea scale (mMRC)), and frequent comorbidities as predictors of PHQ-9 results, while including age, gender, body mass index (BMI) and smoking habits as covariates.

Symptoms and exacerbation risk were associated with depression in an additive manner, with mean elevations in the PHQ-9 sum score by 2.75 and 1.44 points, respectively. Asthma, sleep apnoea, gastrointestinal disorders, osteoporosis and arthritis were linked to increases by 0.8 to 1.3 points. Overall, the COPD characteristics contributed to the mean PHQ-9 score by increases from 4.5 or 5.2 to 6.3 points, respectively, when either taking GOLD A as reference or the absence of comorbidities. This finding was independent of the diagnosis of mental disorder or the intake of antidepressants. The presence of COPD led to an increase in the proportion of scores indicating depression from 12 to 22%. Single item analysis revealed homogenous effects regarding GOLD groups, but heterogeneous effects regarding GOLD grades.

These findings indicate specific effects of COPD severity on the PHQ-9 depression score, especially symptoms and exacerbation risk, explaining the high prevalence of depression in COPD. Alternative explanations like an overlap of COPD severity and PHQ-9 items are discussed. Of note, we also found COPD treatment effects on depression scores.

**Electronic supplementary material:**

The online version of this article (10.1186/s12931-019-0997-y) contains supplementary material, which is available to authorized users.

## Background

Chronic obstructive pulmonary disease is associated with a variety of comorbidities [1, 2], that are associated with hospital admissions and mortality [3]. One of them is depression [4] which is also linked to the clinical state and course of COPD [5, 6]. A further common factor in both COPD and depression are deteriorations in quality of life [7–10].

One of the screening tools for depression is the Patient Health Questionnaire Depression Scale 9, a 9-item self-report module containing the 9 signs and symptoms of major depression as delineated in the Diagnostic and Statistical Manual of Mental Disorders (DSM-IV) [11]; a detailed description of the PHQ-9 questionnaire is given in the Additional file 1. Although the questions were constructed to refer to the same psychometric dimension “depression”, they are not synonymous, and some of them appear to be susceptible to impairments common in somatic diseases.

A relationship between biomedical disease conditions and depression scores has been assessed for a number of chronic diseases [12–14]. In COPD, many studies evaluated the sum score, and some studies selected single items, such as suicide ideation [15]. The spectrum of alterations common in COPD raises the question whether other items of the PHQ-9 and its sum score are related to different COPD characteristics.

To our knowledge, there is no study addressing this question and quantifying the impact of specific COPD characteristics on depression. This impact could also explain the wide range of prevalence estimates of depression in different COPD populations [16, 17]. Based on these considerations, we analysed the relationship between COPD characteristics and the results of the PHQ-9 using data from the baseline visit of the large German COPD cohort COSYCONET (German **CO**PD and **Sy**stemic **Co**nsequences - Comorbidities **Net**work) [18]. To elucidate the impact of COPD, we also demonstrated the associations between the single PHQ-9 items with anthropometric data, gender, age, smoking status, comorbidities and COPD characteristics.

## Methods

### Study population

COSYCONET is a multicentre cohort study focussing on the relationship between the lung disorder and comorbidities. We used the data of the baseline visit comprising 2741 COPD patients; study population, protocol and methods have been described previously [18]. The assessments included functional tests, clinical history, medication, quality of life and the PHQ-9 questionnaire for the screening of depression [18].

Assessments evaluated in the present analysis.

In the present analysis we included all patients of spirometric grades GOLD 1–4 [19], who had complete data of the PHQ-9, spirometric lung function, smoking status (active smoking versus no active smoking), exacerbation history and mMRC. Active smoking was defined as regular or occasional active cigarette smoking within the last 4 weeks prior to the interview. No active smoking included former smokers defined as regular or occasional active cigarette smoking in the past, as well as never smokers defined as no active cigarette smoking over lifetime. For the categorisation into GOLD groups A-D [19] we used the mMRC, which resulted in a more uniform distribution compared to the COPD Assessment Test (CAT), particularly regarding group C [20, 21]. The mMRC was therefore used throughout the study. The definition of exacerbation risk also followed GOLD recommendations [19]. Symptoms were defined via the combined GOLD groups B and D (GOLD BD), and exacerbation risk via the combined GOLD groups C and D (GOLD CD).

Among lung function parameters, forced expiratory volume in one second (FEV_1_) was used, since all other parameters turned out to be redundant for the purpose of this analysis. Predicted values of FEV_1_ were taken from the Global Lung Initiative (GLI) [22]. The assessments including patients’ history, lung function, questionnaires regarding the clinical state of COPD (mMRC, CAT), as well as quality of life (EQ VAS) were guided by standard operating procedures [18], and the PHQ-9 was administered following the instructions [23]. Moreover, patients were asked to bring with them all medication taken, which was recorded and evaluated according to substance categories based on the ATC code [21, 24]. The information derived from medication was also used to define a number of comorbidities for which specific medication was available [24]. The diagnosis (named “extended”) comprised patients with a reported doctor’s diagnosis (anamnestic report) as well as patients without report but with disease-specific medication. For psychopharmacological treatment, we additionally evaluated patients with specific medication and the subset of patients with depression-specific medication.

### Data analysis

Data and parameter estimates are presented as mean values, standard deviations (SD) or standard errors of mean (SEM), or median values and quartiles (mMRC), or numbers and percentages. The relationship between categorical variables was assessed by contingency tables and chi-square statistics. Regarding continuous parameters, analysis of variance (ANOVA) with post hoc-comparisons according to Duncan was used. The individual items of the PHQ-9 were compared between different conditions, e.g. GOLD groups A to D, by the Kruskal-Wallis-H test. The relationship between the PHQ-9 sum score and influencing factors was quantified by multiple linear regression analysis. Additionally, receiver operator characteristics (ROC) were used to evaluate the relationship of the CAT score and EQ VAS to the PHQ-9 score. Statistical significance was assumed for a *p* value below 0.05, and all analyses were performed by the software package SPSS statistics 23 (IBM Corp., Armonk, NY, USA).

## Results

### Study population

Overall, 2291 patients of GOLD grades 1 to 4 were eligible. Of these, 2255 had complete data of the PHQ-9 and GOLD ABCD grouping as well as smoking status and were included into the analysis (Table 1). GOLD grades 1 to 4 were significantly different in the majority of parameters, except for pack years and gender. The distribution of comorbidities over grades and groups is shown in Table 2, indicating that the relative frequency of some comorbid conditions differed across these categories.

**Table 1:** Patients characteristics

	GOLD grades				*p* value	GOLD groups based on mMRC for differentiation between A/C and B/D				*p* value	total (*n* = 2255)
	GOLD 1 (=203)	GOLD 2 (*n* = 948)	GOLD 3 (*n* = 863)	GOLD 4 (*n* = 241)		GOLD A (*n* = 871)	GOLD B (*n* = 567)	GOLD C (*n* = 294)	GOLD D (*n* = 523)		
Gender (m/f)	122/81	573/375	525/338	156/85	0.657	548/323	352/215	175/119	301/222	0.212	1376/879
Age (y) ± SD	66.1 ± 8.7	65.7 ± 8.4	65.0 ± 8.2	62.2 ± 7.9	< 0.001*	65.1 ± 8.5	66.2 ± 8.1	63.7 ± 9.0	64.6 ± 8.0	< 0.001*	65.1 ± 8.4
BMI (kg/m^2^)	26.7 ± 4.6	27.4 ± 5.1	26.5 ± 5.4	24.4 ± 5.0	< 0.001*	26.2 ± 4.6	27.5 ± 5.6	25.8 ± 4.5	27.0 ± 6.0	< 0.001*	26.7 ± 5.2
Pack years	44.8 ± 31.2	50.9 ± 37.5	48.4 ± 34.8	48.5 ± 33.5	0.156	47.6 ± 34.4	53.0 ± 36.9	45.0 ± 33.1	49.9 ± 36.8	0.009*	49.1 ± 35.5
Smoking status active /not active	62/141	271/677	188/675	35/206	< 0.001*	260/581	130/437	73/221	93/430	< 0.001*	556/1699
FEV_1_ (% predicted)	88.7 ± 8.1	62.6 ± 8.3	40.7 ± 5.6	24.8 ± 3.9	< 0.001*	61.3 ± 18.0	47.6 ± 16.6	54.2 ± 16.9	42.4 ± 15.2	< 0.001*	52.5 ± 18.6
CAT	14.2 ± 6.8	16.9 ± 7.1	19.4 ± 7.2	22.1 ± 6.8	< 0.001*	13.9 ± 6.2	20.3 ± 6.2	16.7 ± 6.3	23.7 ± 6.3	< 0.001*	18.1 ± 7.4
mMRC	1.0 ± 0.7	1.3 ± 0.8	1.9 ± 0.9	2.3 ± 0.9	< 0.001*	0.9 ± 0.4	2.4 ± 0.5	0.9 ± 0.4	2.5 ± 0.6	< 0.001*	1.6 ± 0.9
EQ-5D-VAS	67.2 ± 16.9	61.6 ± 18.0	52.7 ± 18.0	46.7 ± 17.0	< 0.001*	67.0 ± 16.8	50.3 ± 16.1	62.4 ± 15.4	45.2 ± 16.3	< 0.001*	57.2 ± 18.8
PHQ-9 Sum score	5.6 ± 4.2	6.0 ± 4.5	6.5 ± 5.0	7.0 ± 5.1	0.002*	4.5 ± 3.7	7.1 ± 4.8	5.9 ± 4.1	8.6 ± 5.2	< 0.001*	6.3 ± 4.7
PHQ-9 ≥ 10 (major depression)%	33 (16.3)	182 (19.2)	201 (23.3)	68 (28.2)	0.002*	88 (10.1)	151 (26.6)	45 (15.3)	200 (38.2)	< 0.001*	484/1771

The table shows mean values (± standard deviations), for gender, smoking status and PHQ-9 values≥10 absolute numbers (percentages). The comparisons of age, BMI, smoking history in terms of pack-years and lung function between GOLD 1–4 and A-D were performed by unadjusted analysis of variance; gender was compared by the chi-squared statistics. *BMI* body-mass index, *FEV*_*1*_ forced expiratory volume in 1 s, *CAT* COPD assessment test, *mMRC* modified Medical Research Council scale, smoking status = active smoking was defined as regular or occasional active cigarette smoking within the last 4 weeks prior to the interview, not active as smoking included former smokers defined as regular or occasional active cigarette smoking in the past, as well as never smokers defined as no active cigarette smoking over lifetime *p < 0.05. For GOLD grades 1–4 the *p*-values refer to the comparisons of all four grades, for groups A-D the *p*-values refer to the comparisons of all four groups

**Table 2:** Distribution of comorbidities over grades and groups

	GOLD grades				*p* value	GOLD groups				*p* value	total (n = 2255)
	GOLD 1 (*n* = 203)	GOLD 2 (*n* = 948)	GOLD 3 (*n* = 863)	GOLD 4 (*n* = 241)		GOLD A *(n* = 871)	GOLD B (*n* = 567)	GOLD C (*n* = 294)	GOLD D (*n* = 523)		
Asthma (ext.) (%)	39 (19.2)	186 (19.6)	169 (19.6)	32 (13.3)	0.135	137 (15.7)	92 (16.2)	78 (26.5)	119 (22.8)	< 0.001*	426/1829
Sleep Apnea (%)	26 (12.8)	102 (10.8)	93 (10.8)	17 (7.1)	0.230	66 (7.6)	66 (11.6)	29 (9.9)	77 (14.7)	< 0.001*	238/2017
Diabetes (ext) (%)	22 (10.8)	130 (13.7)	114 (13.2)	28 (11.6)	0.637	95 (10.9)	76 (13.4)	31 (10.5)	92 (17.6)	0.002*	294/1961
Hyperlipidemia (ext) (%)	104 (51.2)	432 (45.6)	360 (41.7)	72 (29.9)	< 0.001*	381 (43.7)	224 (39.5)	126 (42.9)	237 (45.3)	0.244	968/1287
Hyperuricemia (ext) (%)	31 (15.3)	183 (19.3)	159 (18.4)	33 (13.7)	0.154	123 (14.1)	114 (20.1)	64 (21.8)	105 (20.1)	0.002*	406/1849
Gastrointestinal disorders (ext) (%)	111 (54.7)	431 (45.5)	388 (45.0)	110 (45.6)	0.084	339 (38.9)	257 (45.3)	140 (47.6)	304 (58.1)	< 0.001*	1040/1215
Hypertension (ext) (%)	103 (50.7)	534 (56.3)	516 (59.8)	126 (52.3)	0.041	461 (52.9)	336 (59.3)	165 (56.1)	317 (60.6)	0.020*	1279/976
Coronary Heart Disease (ext) (%)	36 (17.7)	163 (17.2)	146 (16.9)	43 (17.8)	0.984	114 (13.1)	117 (20.6)	39 (13.3)	118 (22.6)	< 0.001*	388/1867
Myocardial Infarction (%)	13 (6.4)	81 (8.5)	82 (9.5)	13 (5.4)	0.153	65 (7.5)	58 (10.2)	19 (6.5)	47 (9.0)	0.162	189/2066
Heart failure (%)	8 (3.9)	45 (4.8)	50 (5.8)	13 (5.4)	0.633	24 (2.8)	31 (5.5)	16 (5.4)	45 (8.6)	< 0.001*	116/2139
Osteoporosis (ext) (%)	29 (14.3)	127 (13.4)	152 (17.6)	56 (23.2)	0.001*	101 (11.6)	94 (16.6)	43 (14.6)	126 (24.1)	< 0.001*	364/1891
Arthritis (%)	21 (10.3)	78 (8.2)	74 (8.6)	12 (5.0)	0.193	67 (7.7)	52 (9.2)	27 (9.2)	39 (7.5)	0.622	185/2070
Mental disorders (anamnestic report) (%)	43 (21.2)	197 (20.8)	166 (19.2)	57 (23.7)	0.491	140 (16.1)	122 (21.5)	60 (20.4)	141 (27.0)	< 0.001*	463/1792

The table shows the distribution of the different comorbidities according to GOLD grades 1–4 and GOLD groups A-D. The comparisons were performed by the chi-squared statistics, significant differences are marked with * *p* < 0.05

### Relationship between PHQ-9 and COPD severity

**Fig. 1:**
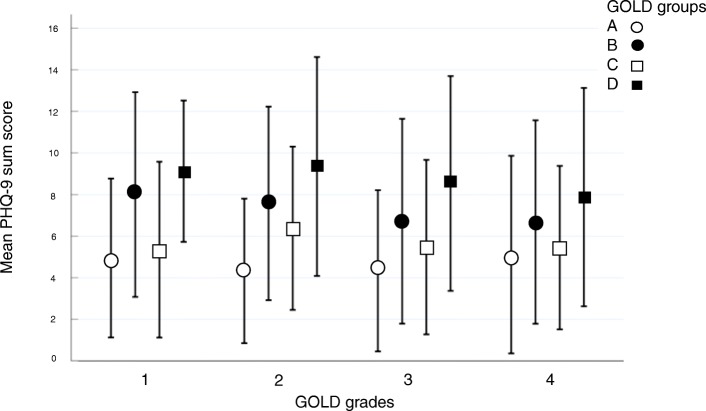
Distribution of the PHQ-9 sum score stratified according to GOLD grades 1–4 and groups A-D in terms of mean PHQ-9 sum score and standard deviation. To illustrate the variability across patients, standard deviations have been chosen. The figure illustrates the additivity of symptoms and exacerbation risk regarding the sum score, when comparing the changes from A to B and B to D with those from A to C and B to D

Multiple linear regression analysis revealed that the sum score of the PHQ-9 was associated with gender, age, BMI, pack years and smoking status (*p* < 0.05 each). The different role of the single of the single items of PHQ-9 to gender, age, BMI, pack years and smoking status is illustrated in Additional file 2: Figure S1, indicating different associations of these data with single PHQ-9 items. For example, higher age was associated with a decrease in the PHQ-9 scores for all questions, in contrast to gender, which was related to an increase of PHQ-9 sum score especially for questions 3 and 5. The distribution of the PHQ-9 sum score stratified according to GOLD grades and GOLD groups is shown in Table 1 and illustrated as combined relationship in Fig. 1, indicating a larger variation across groups than across grades. The figure also demonstrates that the differences related to symptoms (GOLD B and D) were larger than those related to exacerbation risk (GOLD C and D). With increasing severity of COPD the proportion of patients showing scores of at least 10, i.e. depression according to PHQ-9 recommendations, increased (Table 1).

**Fig. 2:**
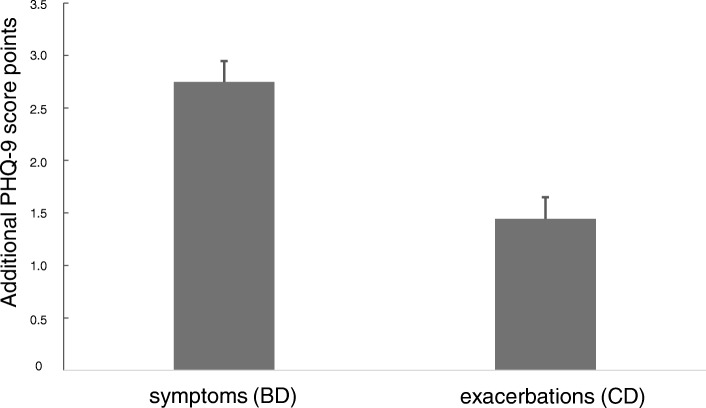
Effect of symptoms and exacerbations on the PHQ-9 sum score. The figure depicts the amounts of the additive effects, corresponding to a mean increase by 2.75 points in patients with high symptom burden and by 1.44 points in patients with high exacerbation risk

The pattern exhibited in Fig. 1 suggested a dependence of the scores on symptoms and exacerbation risk in an additive manner. To evaluate this hypothesis we performed a two-way ANOVA with “symptoms” and “exacerbations” as binary categorical factors and an interaction term, keeping the confounders age, gender, BMI, smoking status and pack years. Higher symptoms (BD vs AC) led to an average (± SEM) increase by 2.75 ± 0.20 points, and higher exacerbations (CD vs AB) were associated with an average increase by 1.44 ± 0.21 points, without significant interaction (Fig. 2). This additive effect implied an increase by 4.19 points in group D compared to group A.

In a next step, we determined the relationship between GOLD groups A to D and the single items of the PHQ-9 (Kruskal-Wallis-H test). Each item showed significant associations with GOLD groups (*p* < 0.001). Additional file 2: Figure S2 illustrates the homogeneity across items, justifying the analysis of the sum score with regard to groups A to D.

### Association of PHQ-9 with functional and clinical state of COPD

The association of the PHQ sum score with GOLD grades 1 to 4 was significant but weak (Table 1). The result for the single items is illustrated in Additional file 2: Figure S3; questions 1, 2, 4, 8 and 9 (*p* ≤ 0.011 each) were dependent on grades 1 to 4, while other questions were not (Kruskal-Wallis-H test). This demonstrates that the relationship to GOLD grades was less linear than the relationship to GOLD groups.

To elucidate the relation to GOLD grades, a linear regression analysis of the sum score against FEV_1_, including age, gender, BMI, smoking status and pack years as confounders, was performed. Overall, FEV_1_ was significantly (*p* < 0.001) related to the PHQ-9 score (Additional file 3: Table S1) but inspection of Fig. 1 revealed that for each of the GOLD groups A to D the association with FEV_1_, or equivalently GOLD grades, was weak. This was confirmed by separate analyses for each group A-D, in which FEV_1_ was not statistically significant. Due to the inconsistency of results, we therefore decided to omit lung function from the further analyses.

An analogous computation using the CAT and EQ VAS scores showed both to be significantly (*p* < 0.001 each) linked to the PHQ-9 sum score (Additional file 3: Table S2). The close relationship was underlined by ROC analyses, taking a value of PHQ-9 ≥ 10 as indicator for depression as outcome variable. These analyses yielded an area under the curve (AUC) of 0.81 with a cut-off of 20 for CAT, and of 0.75 with cut-off values of about 50 for VAS (total scale 0–100). Based on the very close relationship between the tests, CAT and EQ VAS were also omitted from the further analyses.

### Association of PHQ-9 with comorbidities

**Fig. 3:**
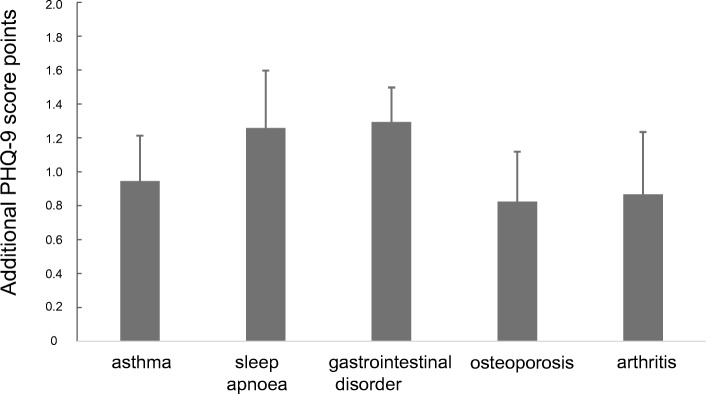
Effect of comorbidities on the PHQ-9 sum score The figure illustrates the dependence of the PHQ-9 score on the different comorbidities. Each single comorbidity was associated with an increase in the PHQ-9 sum score and these effects were additive.

Using multiple linear regression analysis and again including the confounders gender, age, BMI, smoking status and pack years, we assessed the association of the sum score with the comorbidities listed in Table 2. Asthma, sleep apnoea, gastrointestinal disorders, osteoporosis and arthritis were significantly linked to the sum score (Additional file 3: Table S3). The magnitude of the effects is illustrated in Fig. 3. The result for the single items is given in Additional file 1: Figure S4, showing different associations of comorbidities with single PHQ-9 items. For example, sleep apnoea was predominantly related to questions 4 and 7, whereas arthritis was related to questions 1 and 3.

### Association with the diagnosis of mental disorders and medication

To determine the relationship to the PHQ-9 score, different definitions of mental disorders were used (Table 3), again by regression analyses including age, gender, BMI, smoking status and pack years. All four categorisations were significantly related to the PHQ-9 score (*p* < 0.001 each). Intake of specific medication for mental disorders was associated with a mean (±SEM) increase by 2.73 ± 0.31 points, intake of antidepressants with an increase by 2.83 ± 0.33 points, and a reported diagnosis (lifetime) with an increase by 3.94 ± 0.24 points; all associations confirm the suitability of the PHQ-9 to assess effects of medication.

**Table 3:** Different definitions of mental disorders and medication

		reported diagnosis (with/without medication)		total
		No	Yes	
specific medication for mental disorders	No	1708	277	1985 (88.0%)
	Yes	84	186	270 (12.0%)
antidepressants only	No	1731	291	2022 (89.7%)
	Yes	61	172	233 (10.3%)
specific medication and/or reported diagnosis (“extended”)	No	1708	0	1708 (75.7%)
	Yes	84	463	547 (24.3%)
total		1792 (79.5%)	464 (20.5%)	2257

The table shows the distribution of different definitions of mental disorders and their respective medication. The table shows absolute numbers and percentages

### Summary effect of COPD characteristics on the PHQ-9

**Fig. 4:**
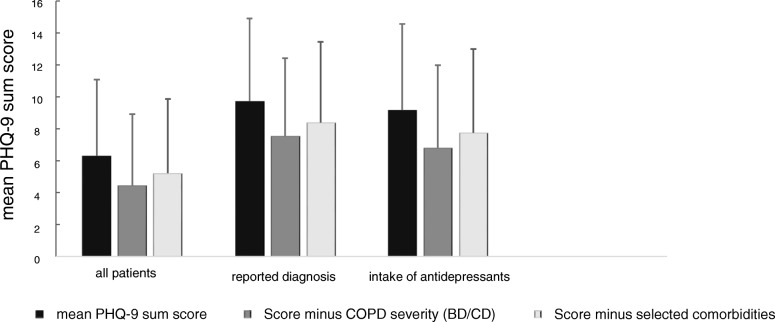
Association of the PHQ-9 sum score to different definitions of mental disorders. This figure illustrates the mean PHQ-9 sum score (black bar), the mean PHQ-9 sum score adjusted for the effect of COPD severity (dark grey bar) and the mean PHQ-9 sum score adjusted for the effect of comorbidities (light grey bar) for all patients (*n* = 2096), for patients with the reported diagnosis of mental disorders (*n* = 464) and for patients with the intake of anti-depressants (*n* = 233). The figure shows that, independent of the subpopulation chosen for analysis, the procedure to take into account COPD characteristics via symptoms/exacerbation risk or comorbidities leads to about the same reduction of the observed PHQ-9 sum score. Moreover it illustrates that both, before and after adjustment, patients with antidepressant therapy showed scores between 6.8 and 9.2, which were larger than those reported in the literature in patients without COPD. This suggests that the adjustment did not fully account for all COPD characteristics, considering the fact that the GOLD group A and the absence of comorbidities were the reference

The observed associations with COPD characteristics raised the question of their overall impact on the PHQ-9 sum score in each individual patient. To quantify this impact, using either COPD severity (groups) or comorbidities as predictors, the two regression models described above were used. Correspondingly, the reference was either the lowest degree of symptoms and exacerbation risk, i.e. GOLD group A, or the absence of all comorbidities shown in Fig. 3. At the same time each patients’ individual values of age, gender, BMI, smoking status and pack years were maintained to account for these influencing factors. Figure 4 illustrates the results, taking the observed mean (±SEM) value of 6.31 ± 0.10 for comparison and demonstrating that in the hypothetical absence of COPD the estimated mean values would be 4.52 ± 0.10 and 5.23 ± 0.10, respectively. This result illustrates the summary effect of COPD in our population on the PHQ-9 score, corresponding to an increase from 12 to 22% regarding patients with PHQ-9 values ≥10 and based on GOLD A-D. The inclusion of patients with a previous diagnosis of mental disorder or intake of antidepressants did not add incremental information to this association.

## Discussion

In the present study, we analysed the impact of COPD on the PHQ-9 depression score to reveal whether specific COPD characteristics were associated with changes in the score. The characteristics identified as relevant for the PHQ-9 comprised symptoms and exacerbation risk according to GOLD groups A to D, and the comorbidities asthma, sleep apnoea, gastrointestinal disorders, osteoporosis and arthritis. The GOLD groups were related to all single PHQ-9 items in a parallel manner, whereas the spirometric GOLD grades 1–4 were only related to some questions. The different role of the single items was underlined by their associations with anthropometric data and comorbidities. Our observations suggest that on average the chronic disease COPD was associated with PHQ-9 scores elevations by 1–2 score points. Conversely, when hypothetically assuming patients being in GOLD group A, or without the comorbidities analysed, the PHQ-9 sum score was reduced by this amount.

In all analyses age, gender, BMI, smoking status and pack years were carried as covariates to adjust for their influences. Females, younger subjects and active smokers showed higher scores of depression [25], which is in line with previous data [26–28]. We additionally found pack years to be related to the sum score, as well as BMI [29]. The associations with age, gender, BMI and smoking were robust throughout all analyses, thereby underlining the validity of the analyses regarding COPD characteristics.

Both categorisations according to GOLD [19] were related to the PHQ-9 sum score but the relationship to groups A-D was much more consistent and stronger than that to grades 1–4. Symptoms and exacerbation risk had completely additive effects not only on the sum score, but on all single items. Overall, in GOLD D compared to A, the PHQ-9 sum score was higher by more than 4 points. The strong effect observed for symptoms is in accordance with published data, despite the fact that these were obtained using previous GOLD definitions [30] or the CAT instead of mMRC score [6]. The average PHQ-9 sum score in our population was about 6.3 points and not far from the cut-off value of ≥10 considered as indicative for depression [11]. In view of this, the magnitude of the effects of COPD on the PHQ-9 score indicate a significant impact on the diagnosis of depression, in accordance with elevated prevalence values in the literature [5].

The mean influence of COPD, based on either symptoms/exacerbations or comorbidities, on the mean PHQ-9 sum score could be quantified as difference between 6.3 versus 4.5 or 5.2 points, respectively (Fig. 4). Formal adjustment of the mean PHQ-9 score for COPD severity or comorbidities led to values that are similar to values reported for the general population [31]. Correspondingly, the prevalence of score values ≥10 decreased from 22.0 to 11.6% or 14.6%, respectively, again being closer to the range of the general population [32, 33]. The effect attributable to COPD was similar in patients with or without the diagnosis of mental disorder or intake of antidepressants, although their baseline values were different. This suggests that COPD severity and comorbidities are associated with a systematic additive effect on the PHQ-9, and the relationship of COPD to the PHQ-9 was not limited to patients with premorbid depressive symptoms. The numerical estimates provided by us (Figs. 2 and 3) might be useful to quantify the effect of individual COPD characteristics on the PHQ-9 score.

Depression is recognized as a major condition among COPD comorbidities [34], but there is a wide range of prevalence estimates [5], suggesting a significant dependence on the study population and possibly the diagnostic tools. The PHQ-9 as a diagnostic screening tool has been validated in the general population [35], as well as cohorts with specific morbidities [12, 14], including COPD. Inspection of its single items raises the question, whether they are sensitive to impairments common in COPD, and indeed some of the items have already been analysed in these patients [15]. There is no study, however, which quantified the effect of COPD characteristics on the PHQ-9 in detail.

For this purpose, we focused on those patients’ characteristics, which are easily available in clinical practice. Lung function was omitted from the final analysis, as the associations were inconsistent and weak compared to the other influencing factors. An important observation was that all effects on the sum score arising from GOLD groups or from comorbidities were additive and that there were no significant interactions. This considerably simplified the estimation of the impact of COPD characteristics (see Figs. 2 and 3). The combination of comorbidities resulted in a slightly lower effect compared to that of symptoms and exacerbation risk (Fig. 4), suggesting additional influencing factors that are covered by GOLD groups, but not the comorbidities chosen. The effect of COPD severity on the PHQ-9 might even be larger as GOLD group A was taken as reference.

The relationship between depression scores and CAT, as a measure of COPD severity, is close, and a cut-off value of 10 for the PHQ-9 corresponds to cut-off values of 19 to 21 for the CAT [6]. We similarly observed the best correspondence for a cut-off value of 20. Interestingly, this value is similar to the cut-off value of 18 that has been proposed as superior to the conventional cut-off value of 10 regarding the categorisation of COPD severity [36]. The predictive power of the CAT score regarding depression seems high, but not high enough to replace a proper depression questionnaire such as the PHQ-9. We found similar results for the visual analogue scale (EQ VAS) of the generic quality of life questionnaire EQ-5D [9, 10]. All of these findings are in line with the expectation that quality of life scores are closely linked to depression.

Based on the patients’ medication and reports we defined several categorical indices of mental disorder, which resulted in prevalence estimates ranging between 10.3 and 24.3% (Table 3). This, however, did not affect our findings regarding the impact of COPD, and the associations found in the total population were also present when excluding patients with antidepressants. The observation that COPD, as common feature of our patients, showed such homogeneous effects, underlines its systematic impact on the PHQ-9 score and the evaluation on depression based on this.

Studies on the effect of antidepressants on the PHQ-9 reported scores of about 17 at baseline and of 5 after treatment [31]. In our study population, patients with antidepressants showed mean scores of 9.2, and of 6.8 or 7.8 when hypothetically taking into account COPD. The decrease in the PHQ-9 score was similar to that in patients without antidepressants but higher than in the total study population, indicating that the antidepressant therapy did not normalize the PHQ-9 score in COPD patients. Although there are no reasons to assume that antidepressants should be less effective in COPD compared to other patients [34], this observation suggest that antidepressant therapy is not fully effective in COPD, possibly due to the impact of continuing symptom burden from respiratory disease.

### Limitations

In COSYCONET the presence of mental disorders was asked as lifetime prevalence based on the diagnosis by a physician, and depression was not specifically asked for. However, patients with a diagnosis of depression at the time of the study could be identified by specific medication, and our findings regarding the impact of COPD on the PHQ-9 score were not affected by this. In addition, the effect of respiratory symptoms and exacerbation risk were additive throughout all analyses in a very consistent manner. We cannot exclude selection effects that are unavoidable in a cohort study with comprehensive, demanding assessments, but it seems unlikely that these have affected our results.

The questionnaires used for the diagnosis of depression are not necessarily equivalent [37], and our results strictly apply to the PHQ-9, which refers to the patients’ clinical state in the previous two weeks. This restriction is unlikely to have played a role in our study, as only patients with stable COPD were investigated [18]. The PHQ-9 could be particularly susceptible to COPD severity, as some of its questions, although targeting depression, address symptoms that are also common in COPD. This was reflected in the correlation between the PHQ-9 and the CAT or EQ VAS, which do specifically target depression, as well as the analysis of single PHQ-9 items. It would be of interest to clarify whether other diagnostic tools for depression also reflect COPD characteristics.

## Conclusion

In a large cohort of patients with COPD, there were significant associations between parameters describing the patients‘clinical state and the PHQ-9 that is used for depression screening. The average magnitude of the effect of COPD severity was 1–2 points which accounted for an increase by about factor 2 in the proportion of patients showing PHQ-9 values of at least 10. Noteworthy enough, the approach using GOLD 2017 A to D groups allowed to quantify the impact of COPD on the PHQ-9 score in the most consistent manner, both regarding single items and sum score. The estimates for GOLD groups and comorbidities provided by us may be helpful to assess the effect of COPD in each individual patient. In our study cohort, the patients with antidepressant medication showed an average value of about 9.2 points (see Fig. 4) which is markedly higher than the value of 5.8, which has been reported under treatment with antidepressant medication in the literature [31]. The difference of about 3.4 points corresponds to the combined effects, which we attribute to the symptom burden, exacerbation rates and comorbidities (see Figs. 2 and 3). As exacerbations seem to play a minor role for the PHQ-9 score compared to symptoms and the available bronchodilator therapy in COPD primarily addresses symptoms, it seems plausible that a better treatment of symptoms would also lower the PHQ-9 score. Therefore it might be hypothesized that the optimisation of respiratory therapy in COPD could improve the scoring for depression in COPD patients.

## Additional files

Additional file 1: Supplement Description. (DOCX 16 kb)

Additional file 2: Supplemental Figures. (PPTX 279 kb)

Additional file 3: Supplemental Tables. (DOCX 34 kb)

## Abbreviations

ANOVA: Analysis of variance
AUC: Area under the curve
BMI: Body mass index
CAT: COPD Assessment Test
COPD: Chronic obstructive pulmonary disease
FEV_1_: Forced expiratory volume in 1 s
FVC: Forced vital capacity
GLI: Global Lung Initiative
GLS: Generalized least squares estimation
mMRC: Modified Medical Research Council dyspnoea scale
PHQ-9: Patient Health Questionnaire 9
RMSEA: Root mean square error of approximation (RMSEA)
SD: Standard deviations
SEM: Standard errors of mean

## References

[1] Barnes PJ, Celli BR (2009). Systemic manifestations and comorbidities of COPD. Eur Respir J.

[2] Yin HL, Yin SQ, Lin QY, Xu Y, Xu HW, Liu T (2017). Prevalence of comorbidities in chronic obstructive pulmonary disease patients: A meta-analysis. Medicine (Baltimore).

[3] Divo M, Cote C, de Torres JP, Casanova C, Marin JM, Pinto-Plata V, Zulueta J, Cabrera C, Zagaceta J, Hunninghake G (2012). Comorbidities and risk of mortality in patients with chronic obstructive pulmonary disease. Am J Respir Crit Care Med.

[4] Atlantis E, Fahey P, Cochrane B, Smith S (2013). Bidirectional associations between clinically relevant depression or anxiety and COPD: a systematic review and meta-analysis. Chest.

[5] Matte DL, Pizzichini MM, Hoepers AT, Diaz AP, Karloh M, Dias M, Pizzichini E (2016). Prevalence of depression in COPD: A systematic review and meta-analysis of controlled studies. Respir Med.

[6] Lee YS, Park S, Oh YM, Lee SD, Park SW, Kim YS, In KH, Jung BH, Lee KH, Ra SW (2013). Chronic obstructive pulmonary disease assessment test can predict depression: a prospective multi-center study. J Korean Med Sci.

[7] Zamzam MA, Azab NY, El Wahsh RA, Ragab AZ, Allam EM (2012). Quality of life in COPD patients. Egyptian J Chest Dis and Tuberc.

[8] Jones GL (2016). Quality of life changes over time in patients with chronic obstructive pulmonary disease. Curr Opin Pulm Med.

[9] Wacker ME, Jörres RA, Karch A, Wilke S, Heinrich J, Karrasch S, Koch A, Schulz H, Watz H, Leidl R (2016). Assessing health-related quality of life in COPD: comparing generic and disease-specific instruments with focus on comorbidities. BMC Pulm Med.

[10] Wacker ME, Jorres RA, Karch A, Koch A, Heinrich J, Karrasch S, Schulz H, Peters A, Glaser S, Ewert R (2016). Relative impact of COPD and comorbidities on generic health-related quality of life: a pooled analysis of the COSYCONET patient cohort and control subjects from the KORA and SHIP studies. Respir Res.

[11] Kroenke K, Spitzer RL, Williams JB (2001). The PHQ-9: validity of a brief depression severity measure. J Gen Intern Med.

[12] Khawaja ISW, Joseph J, Gajwani P, Feinstein RE (2009). Depression and coronary artery disease: the association, Mechanisms, and Therapeutic Implications. Psychiatry (Edgmont).

[13] Park SC, Lee HY, Lee DW, Hahn SW, Park SH, Kim YJ, Choi JS, Lee HS, Lee SI, Na KS (2017). Screening for depressive disorder in elderly patients with chronic physical diseases using the patient health Questionnaire-9. Psychiatry Investig.

[14] Janssen EP, Kohler S, Stehouwer CD, Schaper NC, Dagnelie PC, Sep SJ, Henry RM, van der Kallen CJ, Verhey FR, Schram MT (2016). The patient health Questionnaire-9 as a screening tool for depression in individuals with type 2 diabetes mellitus: the Maastricht study. J Am Geriatr Soc.

[15] Fleehart S, Fan VS, Nguyen HQ, Lee J, Kohen R, Herting JR, Matute-Bello G, Adams SG, Pagalilauan G, Borson S (2015). Prevalence and correlates of suicide ideation in patients with COPD: a mixed methods study. Int J Chron Obstruct Pulmon Dis.

[16] Biswas D, Mukherjee S, Chakroborty R, Chatterjee S, Rath S, Das R, Begum S (2017). Occurrence of anxiety and depression among stable COPD patients and its impact on functional capability. J Clin Diagn Res.

[17] Yohannes AM, Alexopoulos GS (2014). Depression and anxiety in patients with COPD. Eur Respir Rev.

[18] Karch A, Vogelmeier C, Welte T, Bals R, Kauczor HU, Biederer J, Heinrich J, Schulz H, Glaser S, Holle R (2016). The German COPD cohort COSYCONET: aims, methods and descriptive analysis of the study population at baseline. Respir Med.

[19] Vogelmeier CF, Criner GJ, Martinez FJ, Anzueto A, Barnes PJ, Bourbeau J, Celli BR, Chen R, Decramer M, Fabbri LM (2017). Global strategy for the diagnosis, management and prevention of chronic obstructive lung disease 2017 report: GOLD executive summary. Respirology.

[20] Kahnert K, Alter P, Young D, Lucke T, Heinrich J, Huber RM, Behr J, Wacker M, Biertz F, Watz H (2018). The revised GOLD 2017 COPD categorization in relation to comorbidities. Respir Med.

[21] Graf J, Jorres RA, Lucke T, Nowak D, Vogelmeier CF, Ficker JH (2018). Medical treatment of COPD. Dtsch Arztebl Int.

[22] Quanjer PH, Stanojevic S, Cole TJ, Baur X, Hall GL, Culver BH, Enright PL, Hankinson JL, Ip MS, Zheng J (2012). Multi-ethnic reference values for spirometry for the 3-95-yr age range: the global lung function 2012 equations. Eur Respir J.

[23] Löwe B, Spitzer RL, Zipfel S, Herzog W: Gesundheitsfragebogen für Patienten (PHQ D). Komplettversion und Kurzform. Testmappe mit Manual, Fragebögen, Schablonen. Pfizer 2002.

[24] Lucke T, Herrera R, Wacker M, Holle R, Biertz F, Nowak D, Huber RM, Sohler S, Vogelmeier C, Ficker JH (2016). Systematic analysis of self-reported comorbidities in large cohort studies - A novel stepwise approach by evaluation of medication. PLoS One.

[25] Cleland JA, Lee AJ, Hall S (2007). Associations of depression and anxiety with gender, age, health-related quality of life and symptoms in primary care COPD patients. Fam Pract.

[26] Busch MA, Maske UE, Ryl L, Schlack R, Hapke U (2013). prevalence of depressive symptoms and diagnosed depression among adults in Germany: results of the German health interview and examination survey for adults (DEGS1). Bundesgesundheitsblatt Gesundheitsforschung Gesundheitsschutz.

[27] Petersen JJ, Paulitsch MA, Hartig J, Mergenthal K, Gerlach FM, Gensichen J (2015). Factor structure and measurement invariance of the patient health Questionnaire-9 for female and male primary care patients with major depression in Germany. J Affect Disord.

[28] Luger TM, Suls J, Vander Weg MW (2014). How robust is the association between smoking and depression in adults? A meta-analysis using linear mixed-effects models. Addict Behav.

[29] Luppino FS, de Wit LM, Bouvy PF. overweight, obesity, and depression A systematic review and meta-analysis of longitudinal studies. Arch Gen Psychiatry. 2010:220–9. 10.1001/archgenpsychiatry.2010.2 20194822

[30] Vestbo J, Hurd SS, Agustí AG, Jones PW, Vogelmeier C, Anzueto A, Barnes PJ, Fabbri LM, Martinez FJ, Nishimura M (2013). Global strategy for the diagnosis, management, and prevention of chronic obstructive pulmonary disease. Am J Respir Crit Care Med.

[31] Löwe B, Schenkel I, Carney-Doebbeling C, Göbel C (2006). Responsiveness of the PHQ-9 to psychopharmacological depression treatment. Psychosomatics.

[32] Zhang M, Ho RC, Cheung M, Fu E, Mak A (2011). Prevalence of depressive symptoms in patients with chronic obstructive pulmonary disease: a systematic review, meta-analysis and meta-regression. Gen Hosp Psychiatry.

[33] Herbig B, Dragano N, Angerer P (2013). Health in the long-term unemployed. Dtsch Arztebl Int.

[34] Hegerl U, Mergl R (2014). Depression and suicidality in COPD: understandable reaction or independent disorders?. Eur Respir J.

[35] Spitzer RL, Kroenke K, Williams JB (1999). Validation and utility of a self-report version of PRIME-MD: the PHQ primary care study. J Am Med Assoc.

[36] Smid DE, Franssen FME, Gonik M, Miravitlles M, Casanova C, Cosio BG, de Lucas-Ramos P, Marin JM, Martinez C, Mir I et al: Redefining Cut-Points for High Symptom Burden of the Global Initiative for Chronic Obstructive Lung Disease Classification in 18,577 Patients With Chronic Obstructive Pulmonary Disease. J Am Med Dir Assoc 2017, 18(12):1097 e1011–1097 e1024. 10.1016/j.jamda.2017.09.003 29169740

[37] Kendrick T, Dowrick C, McBride A, Howe A, Clarke P, Maisey S, Moore M, Smith PW (2009). Management of depression in UK general practice in relation to scores on depression severity questionnaires: analysis of medical record data. BMJ.

