# Supplementary material for: Effect of COPD severity and comorbidities on the result of the PHQ-9 tool for the diagnosis of depression: results from the COSYCONET cohort study
Source: Respir Res. 2019 Feb 11;20:30. doi: 10.1186/s12931-019-0997-y (PMC6371561; doi:10.1186/s12931-019-0997-y)
Supplement: Supplementary file 1 — Supplement Description. (DOCX 16 kb) [file 12931_2019_997_MOESM1_ESM.docx]

**Supplement**

PHQ-9 Questionnaire

(based on Kroenke KS. The PHQ-9 Validity of a brief depression severity measure. 2001)

Over the last 2 weeks, how often have you been bothered by any of the following problems?

1. Little interest or pleasure in doing things?
2. Feeling down, depressed or hopeless
3. Trouble falling or staying asleep, or sleeping too much
4. Feeling tired or having little energy
5. Poor appetite or overeating
6. Feeling bad about yourself – or that you are a failure or have let yourself or your family down
7. Trouble concentrating on things, such as reading the newspaper or watching television
8. Moving or speaking so slowly that other people could have noticed? Or the opposite – being so fidgety or restless that you have been moving around a lot more than usual
9. Thoughts that you would be better of dead or of hurting yourself in some way

Single items of the PHQ-9

Figure S1 shows the effects of anthropometric data on single PHQ-9 items.

Figure S2 shows the dependence on GOLD groups A-D for each of the 9 questions of the PHQ-9, as well as for the sum score divided by 9. F or each of the single questions, the dependence was additive regarding symptoms (GOLD B and D) and exacerbation risk (GOLD C and D). The results of the sum score is shown as rightmost group of data points and should be compared with figure 1, which additionally illustrates the sum score stratified for GOLD grades 1-4.

In an analogous fashion, figure S3 shows the dependence on GOLD grades 1-4 for each of the 9 questions of the PHQ-9, as well as for the sum score divided by 9. From the data it becomes clear that only some of the questions were related to the GOLD grades. As the GOLD grades are more or less equivalent to FEV_1_ %predicted, this heterogeneity was probably one of the reasons why the regression analyses did not yield consistent results for FEV_1_. This prompted us to omit this parameter from the analyses.

Results of the multiple linear regression analyses

Table S1 shows the results of a multiple linear regression analysis of the PHQ-9 sum score comprising age, gender, BMI, smoking status and pack years as well as FEV_1_ %predicted as predictors. The negative regression coefficient for FEV_1_ indicated that with lower FEV_1_ the sum score on average increased, however inspection of figure 1 demonstrates that the dependence on FEV_1_ (GOLD grades) was considerably weaker than that on GOLD groups. Furthermore, for the single GOLD groups there appeared to be no monotonous relationship. This was reflected in the fact that each of the analyses performed for GOLD groups A-D separately did not indicate a significant dependence on FEV_1_ %predicted.

Table S2 presents the regression coefficients of the PHQ-9 sum score obtained for CAT and EQ VAS as predictors, again carrying age, gender, BMI, smoking status and pack years as covariates. There was a strong relationship between the sum score and CAT and EQ VAS, which was underlined by the results of ROC analyses (see main text). Due to this strong relationship, which is understandable particularly when considering the questions of the CAT, we omitted both parameters from our analyses.

Table S3 shows the results obtained for the PHQ-9 sum score when retaining all comorbidities that were significant after a stepwise search for significant predictors, starting with the comorbidities of table 2. Again, age, gender, BMI, smoking status and pack years were included as covariates. The fact that the estimates for these covariates were very similar in tables S1, S2 and S3 underlines the robustness of the estimates referring to the covariates.
