# Supplementary material for: Effect of COPD severity and comorbidities on the result of the PHQ-9 tool for the diagnosis of depression: results from the COSYCONET cohort study
Source: Respir Res. 2019 Feb 11;20:30. doi: 10.1186/s12931-019-0997-y (PMC6371561; doi:10.1186/s12931-019-0997-y)
Supplement: Supplementary file 2 — Supplemental Figures. (PPTX 279 kb) [file 12931_2019_997_MOESM2_ESM.pptx]

## Slide 1
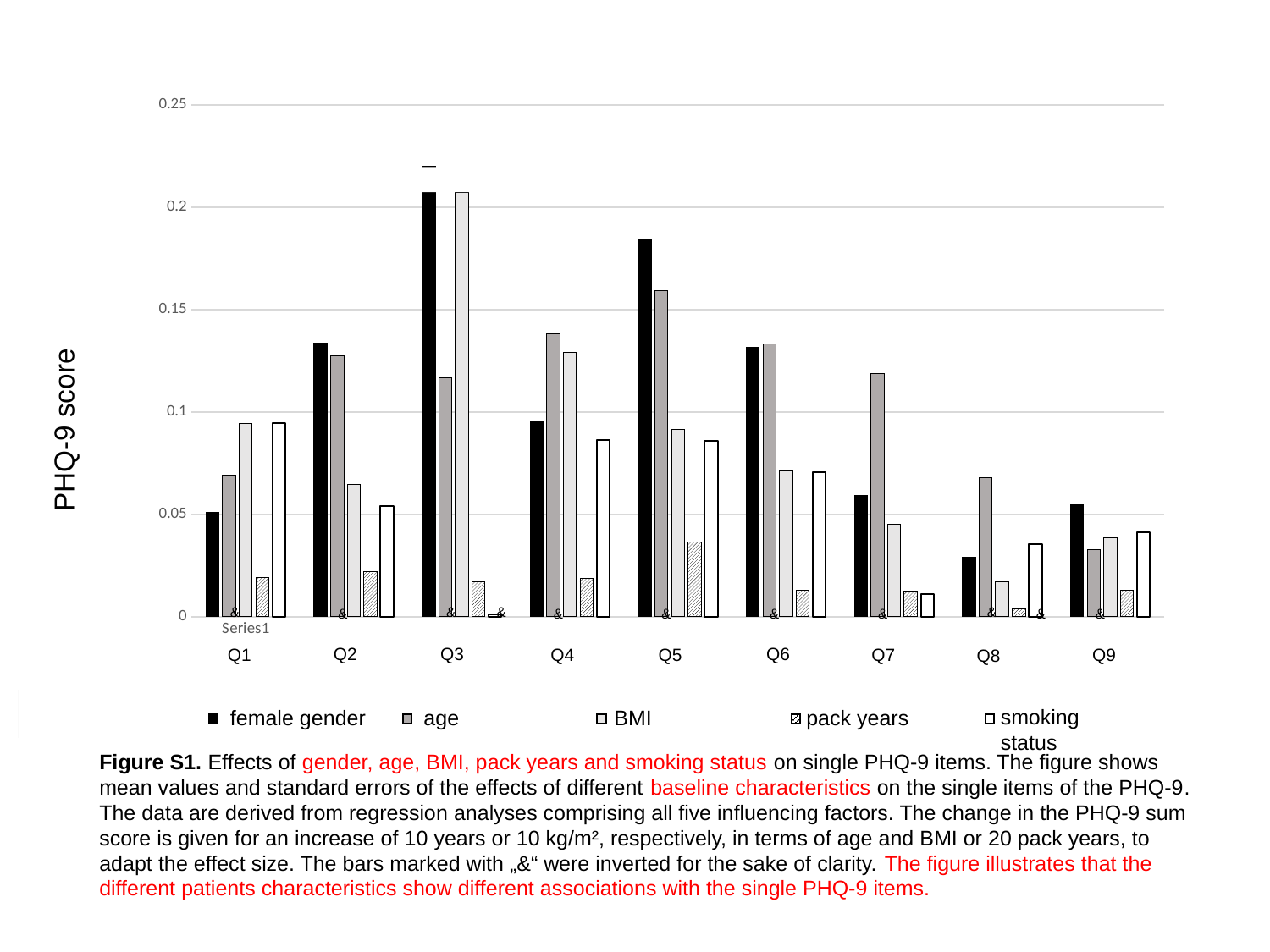

### Chart
| Category | | | | | |
|---|---|---|---|---|---|
| | 0.051117 | 0.06895 | 0.09455 | 0.019139999999999997 | 0.094592 |
| | 0.133599 | 0.12769 | 0.06473999999999999 | 0.02196 | 0.054087 |
| | 0.220087 | 0.11678 | 0.20743 | 0.01708 | 0.001158 |
| | 0.095502 | 0.13832 | 0.12902 | 0.018600000000000002 | 0.086269 |
| | 0.18447 | 0.15925 | 0.09151 | 0.0366 | 0.085848 |
| | 0.131666 | 0.13341 | 0.07118000000000001 | 0.013 | 0.070586 |
| | 0.059394 | 0.11887 | 0.04528 | 0.01236 | 0.010907 |
| | 0.029048 | 0.0677 | 0.01708 | 0.00376 | 0.035312 |
| | 0.055072 | 0.03267 | 0.038489999999999996 | 0.01292 | 0.041154 |
PHQ-9 score
&
&
&
&
&
&
&
&
&
&
&
Q3
Q2
Q6
Q7
Q4
Q5
Q1
Q9
Q8
age
smoking status
pack years
female gender
BMI
Q2
Figure S1. Effects of gender, age, BMI, pack years and smoking status on single PHQ-9 items. The figure shows mean values and standard errors of the effects of different baseline characteristics on the single items of the PHQ-9. The data are derived from regression analyses comprising all five influencing factors. The change in the PHQ-9 sum score is given for an increase of 10 years or 10 kg/m², respectively, in terms of age and BMI or 20 pack years, to adapt the effect size. The bars marked with „&“ were inverted for the sake of clarity. The figure illustrates that the different patients characteristics show different associations with the single PHQ-9 items.

## Slide 2
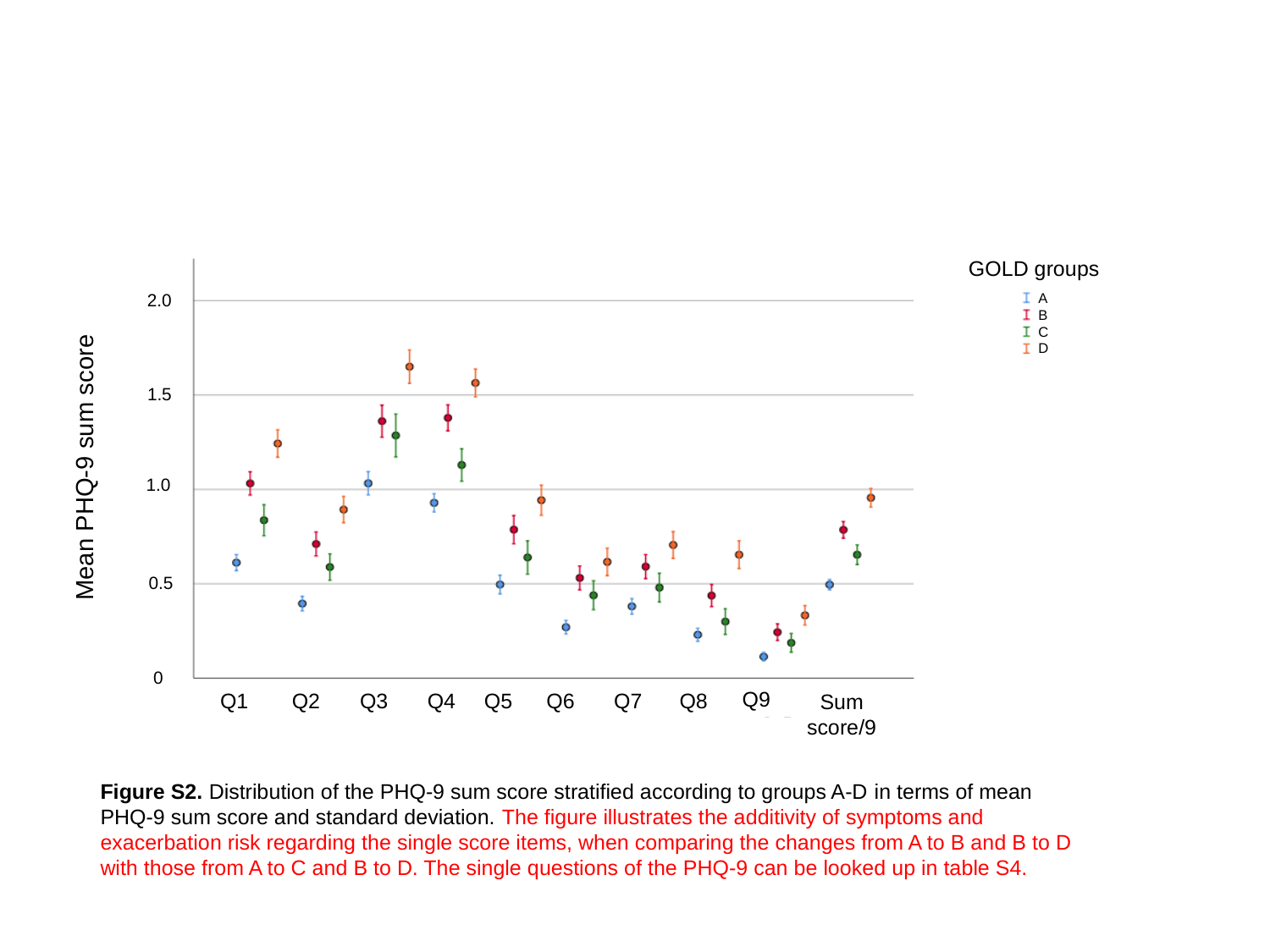

GOLD groups
2.0
A
B
C
D
1.5
1.5
Mean PHQ-9 sum score
1.0
1,0
0.5
0,5
0
0
Q9
Q8
Q7
Q6
Q1
Q2
Q3
Q4
Q5
Sum score/9
Figure S2. Distribution of the PHQ-9 sum score stratified according to groups A-D in terms of mean PHQ-9 sum score and standard deviation. The figure illustrates the additivity of symptoms and exacerbation risk regarding the single score items, when comparing the changes from A to B and B to D with those from A to C and B to D. The single questions of the PHQ-9 can be looked up in table S4.

## Slide 3
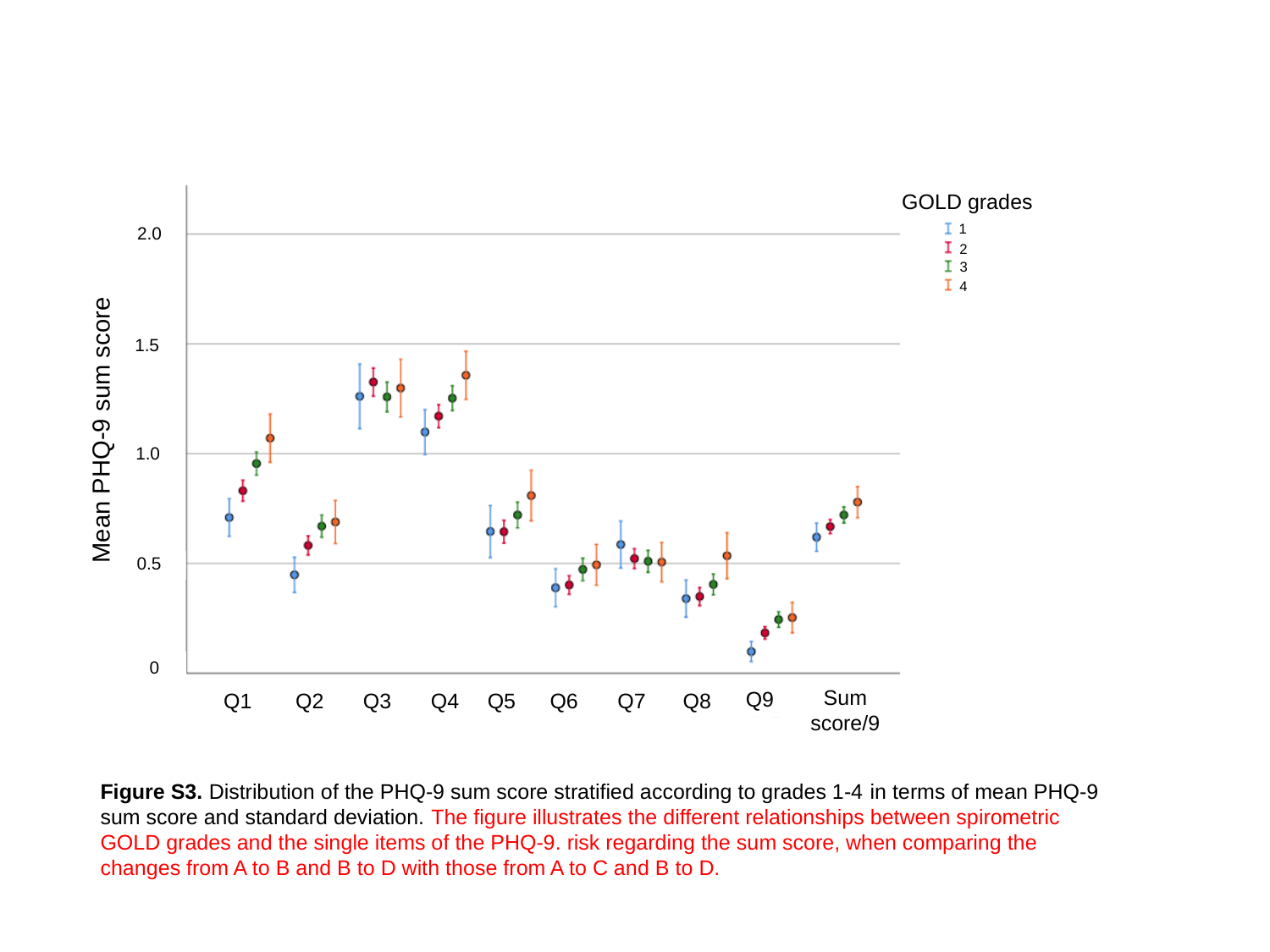

GOLD grades
1
2.0
2,0
2
3
4
1.5
1,5
Mean PHQ-9 sum score
1.0
1,0
0.5
0,5
0
0
Sum score/9
Q9
Q8
Q7
Q6
Q1
Q2
Q3
Q4
Q5
Figure S3. Distribution of the PHQ-9 sum score stratified according to grades 1-4 in terms of mean PHQ-9 sum score and standard deviation. The figure illustrates the different relationships between spirometric GOLD grades and the single items of the PHQ-9. risk regarding the sum score, when comparing the changes from A to B and B to D with those from A to C and B to D.

## Slide 4
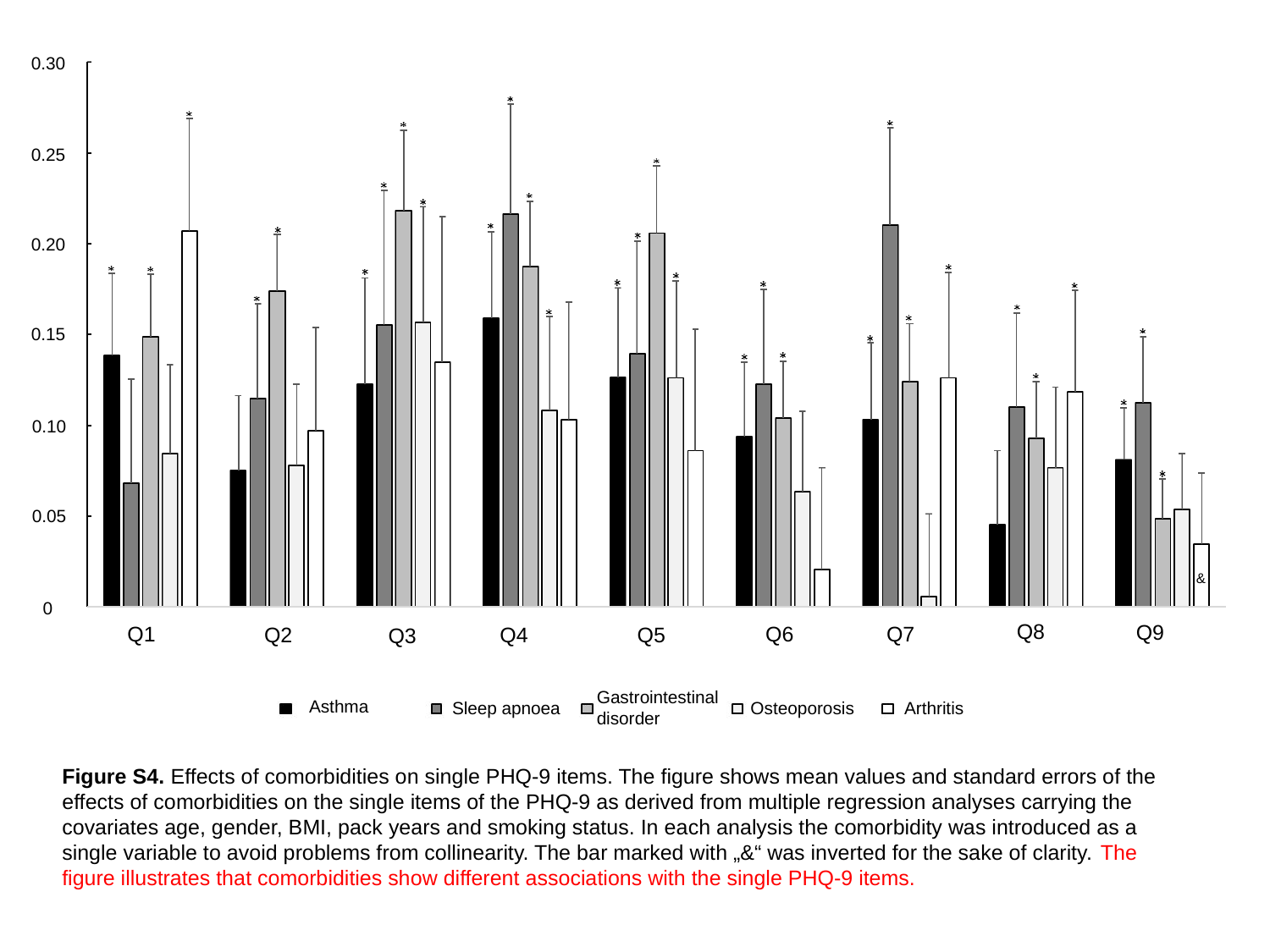

0.30
0.25
0.20
0.15
0.10
0.05
&
0
Q8
Q9
Q7
Q6
Q1
Q5
Q2
Q4
Q3
Gastrointestinal disorder
Asthma
Sleep apnoea
Osteoporosis
Arthritis
Figure S4. Effects of comorbidities on single PHQ-9 items. The figure shows mean values and standard errors of the effects of comorbidities on the single items of the PHQ-9 as derived from multiple regression analyses carrying the covariates age, gender, BMI, pack years and smoking status. In each analysis the comorbidity was introduced as a single variable to avoid problems from collinearity. The bar marked with „&“ was inverted for the sake of clarity. The figure illustrates that comorbidities show different associations with the single PHQ-9 items.
