# Supplementary material for: Effect of COPD severity and comorbidities on the result of the PHQ-9 tool for the diagnosis of depression: results from the COSYCONET cohort study
Source: Respir Res. 2019 Feb 11;20:30. doi: 10.1186/s12931-019-0997-y (PMC6371561; doi:10.1186/s12931-019-0997-y)
Supplement: Supplementary file 3 — Supplemental Tables. (DOCX 34 kb) [file 12931_2019_997_MOESM3_ESM.docx]

| **coefficients^a^** | | | | | | | | |
| --- | --- | --- | --- | --- | --- | --- | --- | --- |
| Modell | | Non-standardized coefficient | | standardized coefficient | T | Sig. | 95 % confidence intervals for B | |
|  |  | Regression coefficient B | Std.-error | Beta |  |  | Lower Limit | Upper limit |
| 1 | (constant) | 18.110 | 1.094 |  | 16.548 | 0.000 | 15.964 | 20.256 |
|  | age | -0.089 | 0.013 | -0.155 | -7.035 | 0.000 | -0.113 | -0.064 |
|  | gender | 1.006 | 0.214 | 0.102 | 4.695 | 0.000 | 0.586 | 1.426 |
|  | BMI | 0.088 | 0.020 | 0.098 | 4.527 | 0.000 | 0.050 | 0.127 |
|  | smoking status | 0.564 | 0.245 | 0.052 | 2.302 | 0.021 | 0.084 | 1.045 |
|  | pack years | 0.008 | 0.003 | 0.059 | 2.624 | 0.009 | 0.002 | 0.014 |
|  | FEV1%pred | -0.026 | 0.006 | -0.101 | -4.628 | 0.000 | -0.037 | -0.015 |

**Table S1. Results of regression analysis of the sum score (PHQ-9) regarding spirometric parameters (GOLD 1-4)**

Table S1 shows the results of a multiple linear regression analysis of the PHQ-9 sum score comprising age, gender, BMI, smoking status and pack years as well as FEV_1_ %predicted as predictors. The negative regression coefficient for FEV_1_ indicated that with lower FEV_1_ the sum score on average increased, however inspection of figure 1 demonstrates that the dependence on FEV_1_ (GOLD grades) was considerably weaker than that on GOLD groups. Furthermore, for the single GOLD groups there appeared to be no monotonous relationship. This was reflected in the fact that each of the analyses performed for GOLD groups A-D separately did not indicate a significant dependence on FEV_1_ %predicted.

| **coefficients^a^** | | | | | | | | |  |
| --- | --- | --- | --- | --- | --- | --- | --- | --- | --- |
| Modell | | Non-standardized coefficient | | standardized coefficient | T | Sig. | 95 % confidence intervals for B | |  |
|  |  | Regression coefficient B | Std.-Error | Beta |  |  | Lower Limit | Upper limit |  |
| 1 | (constant) | 13.319 | 1.021 |  | 13.042 | 0.000 | 11.316 | 15.321 |  |
|  | age | -0.066 | 0.010 | -0.116 | -6.702 | 0.000 | -0.086 | -0.047 |  |
|  | gender | 0.861 | 0.169 | 0.088 | 5.086 | 0.000 | 0.529 | 1.193 |  |
|  | BMI | 0.031 | 0.015 | 0.035 | 2.034 | 0.042 | 0.001 | 0.061 |  |
|  | smoking status | 0.407 | 0.192 | 0.038 | 2.127 | 0.034 | 0.032 | 0.783 |  |
|  | pack years | 0.007 | 0.002 | 0.049 | 2.743 | 0.006 | 0.002 | 0.011 |  |
|  | CAT | 0.329 | 0.014 | 0.509 | 23.376 | 0.000 | 0.301 | 0.356 |  |
|  | VAS | -0.037 | 0.005 | -0.147 | -6.790 | 0.000 | -0.048 | -0.026 |  |
| a. dependent variable: PHQ-9 sum score | | | | | | | | |  |

**Table S2. Results of regression analysis of the PHQ-9 sum score regarding CAT score and quality of life**

Table S2 presents the regression coefficients of the PHQ-9 sum score obtained for CAT and EQ VAS as predictors, again carrying age, gender, BMI, smoking status and pack years as covariates. There was a strong relationship between the sum score and CAT and EQ VAS, which was underlined by the results of ROC analyses (see main text). Due to this strong relationship, which is understandable particularly when considering the questions of the CAT, we omitted both parameters from our analyses.

|  | | | | | | |
| --- | --- | --- | --- | --- | --- | --- |
|  | | | | | | |
| parameter | R Regression coefficient B | Std. Error | T | Sig. | 95 % confidence intervals | |
|  |  |  |  |  | Lower limit | Upper limit |
| constant | 24.621 | 1.110 | 22.182 | 0.000 | 22.444 | 26.798 |
| age | -0.097 | 0.012 | -7.877 | 0.000 | -0.121 | -0.073 |
| male | -0.687 | 0.218 | -3.158 | 0.002 | -1.113 | -0.260 |
| female | 0^a^ |  |  |  |  |  |
| BMI | 0.054 | 0.020 | 2.733 | 0.006 | 0.015 | 0.093 |
| non active smoker | -0.553 | 0.240 | -2.307 | 0.021 | -1.023 | -0.083 |
| active smoker | 0^a^ |  |  |  |  |  |
| pack years | 0.008 | 0.003 | 2.691 | 0.007 | 0.002 | 0.014 |
| no asthma | -0.945 | 0.268 | -3.532 | 0.000 | -1.470 | -0.420 |
| asthma | 0^a^ |  |  |  |  |  |
| no sleep apnoea | -1.251 | 0.339 | -3.691 | 0.000 | -1.915 | -0.586 |
| sleep apnea | 0^a^ |  |  |  |  |  |
| no gastrointestinal disorder | -1.304 | 0.203 | -6.420 | 0.000 | -1.703 | -0.906 |
| gastrointestinal disorder | 0^a^ |  |  |  |  |  |
| no osteoporosis | -0.754 | 0.290 | -2.602 | 0.009 | -1.322 | -0.186 |
| osteoporosis | 0^a^ |  |  |  |  |  |
| no arthritis | -0.860 | 0.366 | -2.349 | 0.019 | -1.578 | -0.142 |
| arthritis | 0^a^ |  |  |  |  |  |

**Table S3. Results of regression analysis of the PHQ-9 sum score retaining all comorbidities**

Table S3 shows the results obtained for the PHQ-9 sum score when retaining all comorbidities that were significant after a stepwise search for significant predictors, starting with the comorbidities of table 2. Again, age, gender, BMI, smoking status and pack years were included as covariates. The fact that the estimates for these covariates were very similar in tables S1, S2 and S3 underlines the robustness of the estimates referring to the covariates.
